# Ankle tuberculosis: an unusual presentation

**DOI:** 10.11604/pamj.2023.45.127.40871

**Published:** 2023-07-17

**Authors:** Hardik Rasiklal Patel, Siddharth Patel

**Affiliations:** 1 Department of Orthopedics, Datta Meghe Institute of Medical Sciences (DU), Sawangi, Wardha, Maharashtra, India

**Keywords:** Ankle tuberculosis, tuberculosis, extrapulmonary tuberculosis, osteoarticular tuberculosis

## Image in medicine

Tuberculosis a leading cause of ill-health. Skeletal tuberculosis is a less common extrapulmonary manifestation of the disease which can cause monoarticular involvement. Isolated involvement of joints encompasses a spectrum of differential diagnoses such as infectious, inflammatory or neoplastic processes, where a high degree of suspicion is necessary for the diagnosis of tuberculous involvement. Here, we report a rare presentation of ankle joint monoarthritis due to tuberculosis without pulmonary involvement. Ankle and foot tuberculosis (AFTB) is very uncommon (<5%) of osteoarticular tuberculosis. Tuberculosis disease (TB) is more common among males than females. A 30-year-old male patient was brought to the orthopedics outpatient department with complaints of pain and discharging sinus in his left ankle for 1 year which was increasing over the period of time, with a history of evening rise of temperature, loss of weight, loss of appetite and no history of trauma. On examination, there was soft tissue swelling on the left ankle, a discharging sinus was present and caseous material was seen oozing from the sinus. The patient was taken in emergency operation for debridement and curettage and caseous material was sent for histopathological examination and culture and sensitivity test which was positive for mycobacterium tuberculosis.

**Figure 1:**
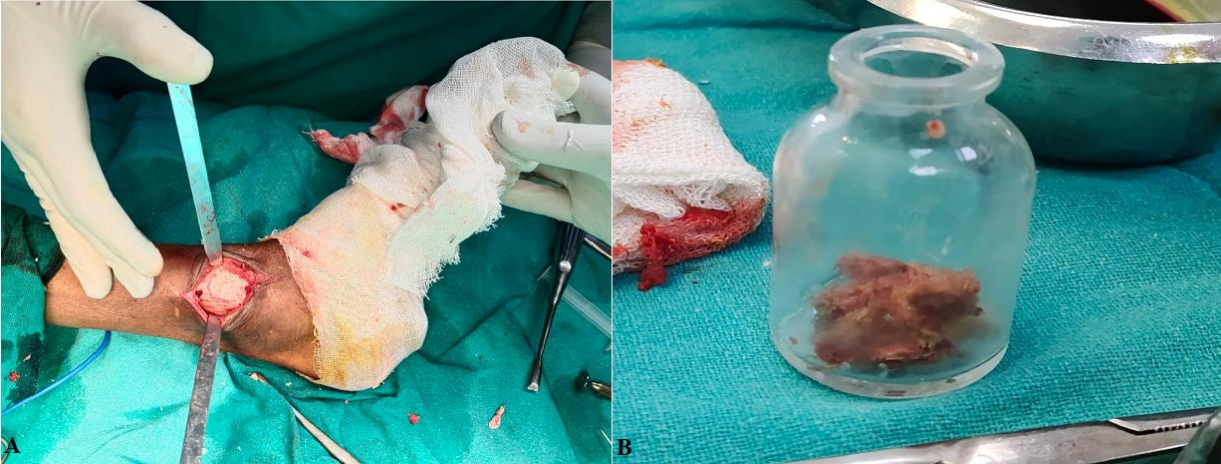
A) intraoperative photograph of caseous material inside ankle joint; B) specimen of caseous material extracted from ankle joint which was sent for culture and sensitivity

